# Portable Gentle Jogger Improves Glycemic Indices in Type 2 Diabetic and Healthy Subjects Living at Home: A Pilot Study

**DOI:** 10.1155/2020/8317973

**Published:** 2020-01-21

**Authors:** Jose A. Adams, Veronica Banderas, Jose R. Lopez, Marvin A. Sackner

**Affiliations:** ^1^Division Neonatology Mt. Sinai Medical Center of Greater Miami, Miami Beach, Florida, USA; ^2^Sackner Wellness Products LLC, Miami, Florida, USA; ^3^Mount Sinai Medical Center of Greater Miami, Miami Beach, Florida, USA; ^4^Medical Services, Mt. Sinai Medical Center of Greater Miami, Miami Beach, Florida, USA

## Abstract

**Background:**

Physical inactivity is a high-risk factor for type 2 diabetes. Increased physical activity improves indices of glycemic control. Continuous glucose monitoring (CGM) allows the investigation of glycemic control during activities of daily living. A pilot study was undertaken to determine the effects of the portable Gentle Jogger (passive simulated jogging device (JD)) that decreases physical inactivity by effortlessly producing body movements on glycemic indices of healthy and type 2 diabetes subjects using CGM during activities of daily living.

**Methods:**

A single-arm, nonblinded study was carried out in 22 volunteers (11 type 2 diabetics and 11 healthy subjects), using continuous glucose monitoring (CGM) for 14 days. On day 4, subjects were provided with JD and instructed to use it a minimum of 3 times per day for 30 min for 7 days. CGM data was analyzed at baseline (BL) and during 2, 3, 4, 5, 6, and 7 days of JD (JD 2, 3, 4, 5, 6, 7) and 1-2-day post JD (Post JD1 and 2) and the following 24 hr indices computed mean glucose (mGLu), SUM of all glucose values, % coefficient of variation (%CV), area under the 24-hour curve (AUC), time spent above range (TAR, glucose 180-250 mg/dl), and time in range (TIR).

**Results:**

In healthy subjects, there were significantly lower values of mGlu and SUM compared to BL for all days of JD usage. In type 2 diabetics, mGlu, SUM, and AUC were significantly lower compared to BL, for all days of JD usage and Post JD1. TAR was significantly lower and TIR significantly improved during JD, in type 2 diabetics without change in %CV.

**Conclusion:**

Gentle Jogger is a portable, passive movement technology that reduces physical inactivity while improving 24 hr glycemic control. It can be self-administered as a standalone device or as an adjunct to diabetic medications. This trial is registered with NCT03550105.

## 1. Introduction

Over 114 million American adults have prediabetes or type 2 diabetes. In terms of economic analysis, total healthcare costs for American diabetics are estimated at $327 billion [1] and on a global basis projected as approximately $2.1 trillion by 2030 [2]. The percentage of adults with diabetes increases with age, reaching a high of 25.2% among those aged 65 years or older. The average cost per person over the age of 65 yr with diabetes is close to $13,000 per year. Risk factor analysis for 2011–2014 of U.S. adults aged 18 years or older with diagnosed diabetes indicated that more than 40.8% of adults were physically inactive [1]. This number is likely to be higher since it accounts for only those defined as getting less than 10 minutes a week of moderate or vigorous activity [3]. In addition to physical inactivity as a risk for diabetes, it is also one of the highest cardiovascular risk factors [4, 5].

Physical inactivity is a growing hazard to health as deduced from activity monitors that showed working adults spend about 60% of their working and waking time engaged in sedentary behavior [6]. The total sitting time for adults in the USA increased from 5.5 hr/day to 6.4 hr/day from 2007 to 2016 [3]. Physical inactivity is associated with cardiovascular disease and all-cause mortality as well as obesity, hypertension, and impaired glucose metabolism [7, 8]. Physical inactivity (sitting or lying still) leads to a rapid onset of rise in blood pressure, decreased nitric oxide bioavailability, and increased sympathetic activity [8–13].

Increasing physical activity of varying duration and intensity improves postprandial glucose handling, long-term glycemic control, time spent in hyperglycemia, and insulin sensitivity in diabetic patients [14–18]. Further, nonexercise physical activity can aid in prevention and treatment of type 2 diabetes [19]. Both sustained hyperglycemia and large glucose fluctuations adversely affect cardiovascular health of diabetic subjects [20].

The American Diabetes Association recommends daily exercise sessions in type 2 diabetics and structured lifestyle interventions which include at least 150 min/week of physical activity as well as dietary changes to delay onset of type 2 diabetes for high-risk individuals and those with prediabetes [4]. In addition to increased physical activity, reducing and regularly interrupting physical inactivity as present in prolonged sitting time is likely to have important and varied benefits across the spectrum of diabetes risk. Unfortunately, achieving these goals is difficult for most individuals to achieve owing to compliance with a behavior change [21].

The advent of continuous glucose monitoring (CGM) has allowed for investigations of glycaemia and the effects of interventions such as pharmacotherapy, diet, and exercise on glycemic control over variable periods of time (hours to weeks). Furthermore, CGM can be performed while the subject interacts with activities of daily living without laboratory constraints, thereby evaluating interventions under real-world conditions [22–24].

The purpose of this pilot study was to determine the daily glycemic effects based on CGM of a self-administered, noninvasive, device called the Gentle Jogger (JD) that provides effortless movements of the lower extremities while sitting from motorized foot pedals.

## 2. Methods

### 2.1. Institutional Review Board Approval

This study and its informed consent were approved by Western Institutional Review Board (WIRB) (WIRB, Puyallup, WA 98374-2115). The study is registered at *ClinicalTrials.gov*NCT03550105. Our study was designed as a nonrandomized single arm pilot study. The study was conducted between May 2018 and March 2019. The inclusion criteria in this protocol were healthy as well as diabetic subjects between the ages of 25 and 85. The diagnosis of type 2 diabetes was determined by their primary physicians and the taking of either insulin or oral diabetic medications. Exclusion criteria included inability to provide informed consent, weight loss surgery, inability to maintain a CGM device during the study period, and lack of compliance with the usage of JD. Subjects were recruited from personal contacts. The study protocol was verbally communicated and all participants signed approved informed consent forms. (Supplemental [Supplementary-material supplementary-material-1]).

### 2.2. Gentle Jogger (JD)

**Figure 1:**
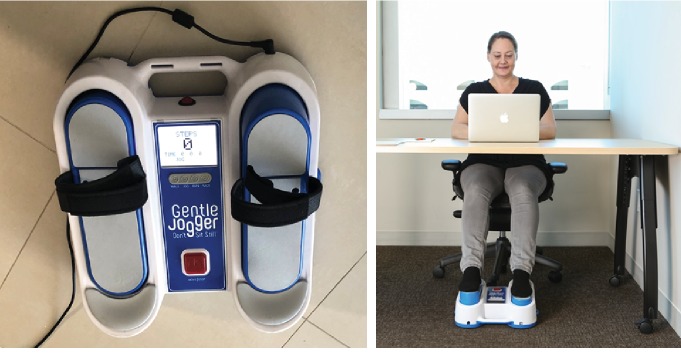
Gentle Jogger (JD). The motorized pedals of the Gentle Jogger repetitively tap against a semirigid surface for simulation of locomotion while subjects are seated or lying in bed.

The portable JD (Sackner Wellness Products LLC, Miami FL33132) incorporates microprocessor-controlled, DC-motorized movements of foot pedals placed within a chassis to repetitively tap against a semirigid surface for simulation of locomotion while the subject is seated or lying in a bed (Figure 1). It weighs about 4.5 kg with chassis dimensions of 34 × 35 × 10 cm. It is placed on the floor for seated applications and secured to the footplate of a bed for supine applications. Its foot pedals rapidly and repetitively alternate between right and left pedal movements to actively lift the forefeet upward about 2.5 cm followed by active downward tapping against a semirigid bumper placed within the chassis. In this manner, it simulates feet impacting against the ground during selective speeds of locomotor activities. Each time the passively moving foot pedals strike the bumper, a small pulse is added to the circulation as a function of pedal speed [25]. Buttons on the chassis offer selection of speeds, viz., walk ~120 steps/min, jog ~150 steps/min, run ~175 steps/min, and race ~190 steps/min. All studies with JD in this paper were done at “race” speed.

### 2.3. Subjects

Twenty-one ambulatory individuals were enrolled and gave their informed consent to participate. Ten subjects were diagnosed as having type 2 diabetes by their primary physician for variable durations of time and were taking insulin or oral diabetic medications. Another 11 subjects in the study were considered “healthy” without prior history of diabetes and had never taken either insulin of oral diabetic medications. There was no attempt to modify diet or physical activity. All subjects received financial remuneration for their participation. BMI was computed to characterize participants as follows: BMI normal weight 18.5 to 24.9, overweight 25 to 29.9, and obese greater than 30 or more. Demographics are shown in Table 1.

On the first day of study, an interstitial continuous glucose monitor (CGM, Free Style Libre Pro, Abbott, Alameda, CA), which provides glucose concentration values every 15 min for 14 days, was fixed over the deltoid area on the nondominant arm. Subjects returned after 2 days and glucose values from the device were reviewed. All volunteers met the following criteria: (a) diabetic subjects had more than 20% of baseline 24 hr glucose values exceeding 150 mg/dl and (b) healthy subjects had median 24 hr glucose < 150 mg/dl.

**Figure 2:**
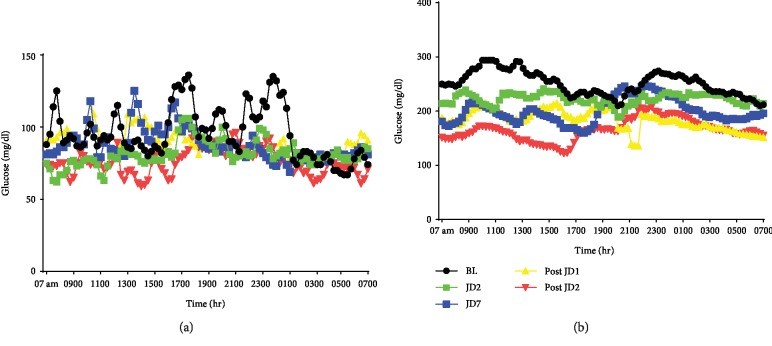
Representative twenty-four-hour glucose values for healthy and diabetic subjects. Twenty-four-hour raw glucose data (mg/dl) obtained from the continuous glucose monitor (CGM) at baseline (BL), 2 and 7 days of JD use (JD2, JD7), and 1 and 2 days after JD use (Post JD1, Post JD2). (a) Healthy subject. (b) Type 2 diabetic subject.

To replicate real-world behavior, subjects were told to continue their current medications without altering dosing or schedule with their same diet and physical activity. Beginning on the morning of day 4 of the study, the subjects were instructed on home usage of JD consisting of at least three times per day for 30 min duration in the “race” mode (approximately 190 pedal steps in place per minute, more than 10,000 pedal steps in place per day in 1 hr). To verify compliance with JD use, they were asked to take pictures of the JD monitoring screen daily with a loaned iPhone and to deliver the iPhone to the study coordinator. Subjects returned JD after 7 days of daily use while continuing CGM until day 14. The CGM sensor was then removed and data exported to an Excel spreadsheet for analysis. Representative raw data from one healthy and one type 2 diabetic are depicted in Figure 2.

### 2.4. Data Analysis

Continuous glucose data recorded every 15 minutes were exported as a text file to an Excel spreadsheet. Data were analyzed in increments of 24 hours starting at 7 am. Data were obtained 24 hr prior to operation of JD (BL), after 2, 3, 4, 5, 6, and 7 days of JD (JD2, JD3, JD4, JD5, JD6, JD7), and 24 and 48 hr after discontinuation of JD (Post JD1, Post JD2). No data points were extrapolated or deleted from the analysis which was performed by comparing each 24 hr data increment to baseline (BL). For each 24 hr period, the mean (mGlu), sum of all glucose data points (SUM), the area under the curve (AUC), and the coefficient of variation (%CV expressed as the mGlu/standard deviation) were computed. The frequency histogram for each 24 hr period was used to calculate the core metrics for percentage of readings and time per day within target glucose range (time in range (TIR), glucose 70-180 g/dl), time above range (TAR, glucose 181-250 mg/dl), and time below range (TBR, glucose < 70 mg/dl) [24]. Since data were not normally distributed, the nonparametric ANOVA Friedman test was used in the analysis. Statistica Software (Statsoft, TIBCO Software Inc., Palo Alto, CA) was used for statistical analyses and plotted on GraphPad Prism 8 (GraphPad Software, San Diego, CA). Data were expressed as the mean ± SD.

## 3. Results

The characteristics of each subject are listed in Table 1. The mean age for healthy subjects was 44.2 ± 16 yr and 60 ± 10.9 yr for diabetics (*p* < 0.02). Two diabetic subjects and two in the healthy group were obese (BMI ≥ 30). One subject claimed to be using JD but did not, all other subjects operated the device as instructed.

### 3.1. Healthy Subjects

**Figure 3:**
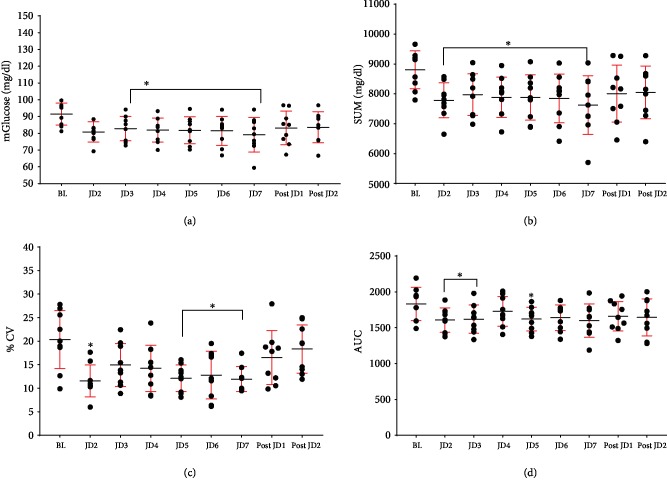
The effects of JD on glycemic indices in healthy adults. Twenty-four-hour data at baseline (BL), 2, 3, 4, 5, 6, and 7 days of jogging device (JD 2, 3, 4, 5, 6, 7) and 1 and 2 days after JD (Post JD 1, 2): (a) mean glucose (mGlu) (mg/dl), (b) 24 hr sum of glucose (SUM), (c) coefficient of variability (%CV), and (d) 24 hr area under the curve (AUC). Each point represents an individual subject, with mean and standard deviations for the group. ^∗^*p* < 0.01 compared to BL.

In healthy subjects, there were significantly lower values from BL of 24 hr mGlu (9 to 12% decrease from BL) and SUM (9-12% decrease from BL) compared to all days of JD usage, but no difference between Post JD1 or Post JD2 and BL. %CV was significantly lower between BL and JD2, JD5, JD6, and JD7, but there was no difference between BL and Post JD1 or 2. AUC was significantly lower between BL and JD2, JD3, and JD5, but did not differ between BL and Post JD1 or 2. There were no glucose values above 180 mg/dl (10 mmol/l) in any of the healthy subjects, and thus, TAR was not reported. Further, there were no glucose values less than 60 mg/dl (3.0 mmol/l) and as expected TIR, TBR were not significantly different between BL and all time periods (Table 2, Figure 3). Post hoc analysis of the above data excluding obese subjects (BMI ≥ 30) also showed the same significant differences (Supplementary [Supplementary-material supplementary-material-1]).

### 3.2. Type 2 Diabetics

**Figure 4:**
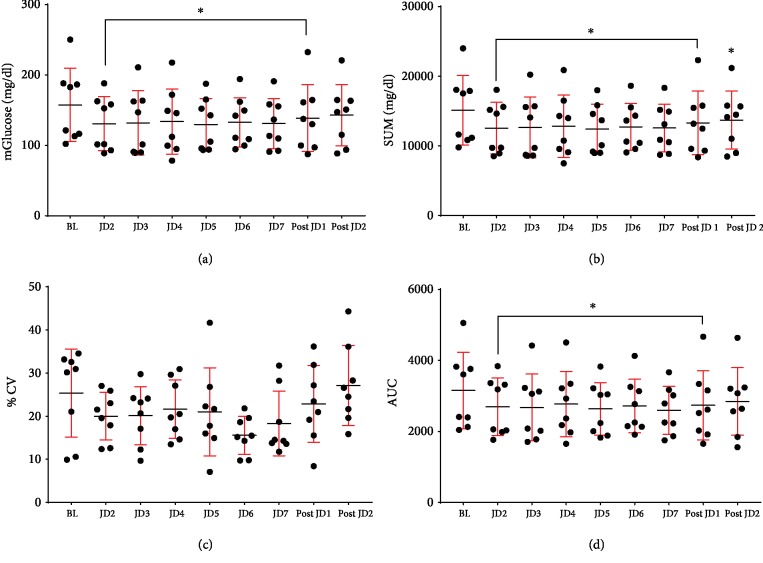
The effects of JD on glycemic indices in type 2 diabetics. Twenty-four-hour data at baseline (BL), 2, 3, 4, 5, 6, and 7 days of jogging device (JD 2, 3, 4, 5, 6, 7) and 1 and 2 days after JD (Post JD 1, 2): (a) mean glucose (mGlu) (mg/dl), (b) 24 hr sum of glucose values (SUM), (c) coefficient of variability (%CV), and (d) 24 hr area under the curve (AUC). Each point represents an individual subject, with the mean and standard deviations for the group. ^∗^*p* < 0.01 compared to BL.

Significantly lower values and differences were found in mGlu ranging from 15 to 19% decrease, SUM ranging from 10 to 19% decrease, and AUC ranging from 11 to 18% decrease from BL compared to all days of JD usage. There was carryover effect for JD as evidenced by a continued decrease in mGlu and SUM, 1 day after discontinuation of JD (Post JD1). The mGlu and SUM decreased by 8 and 10% from BL both 1 and 2 days after JD (Post JD1, Post JD2), respectively. There were no differences in %CV between BL and any JD days or Post JD1 or 2. In all type 2 diabetic subjects, the percentage of time above range (TAR, glucose 181-250 mg/dl) was significantly lower (greater than 50% reduction) between BL and all days of JD usage. There was no difference in TAR between BL and Post JD1 or 2. Time spent in range (TIR) was significantly higher compared to BL during use of JD, but not different after JD. There was no significant difference in time below range (TBR glucose < 70 mg/dl) from baseline during use of JD (Table 3 and Figure 4). Post hoc analysis of the above data excluding obese subjects (BMI ≥ 30) also showed the same significant differences (Supplementary [Supplementary-material supplementary-material-1]).

## 4. Discussion

The major findings in this pilot study provided by the Gentle Jogger (JD) were (i) decreased 24 hr mGlu, total SUM of blood glucose values in both type 2 diabetics and healthy subjects, (ii) reduced AUC and amount of time spent in hyperglycemia in type 2 diabetics, as well as significant improvement in time spent in glucose range (70-180 mg/dl), and (iii) carryover effect of JD for 24 hr in type 2 diabetic subjects for mGlu, SUM, and AUC.

Continuous glucose monitoring (CGM) allows for investigation of real-life conditions of diet and activities of daily living. The portable JD effortlessly produces body movements while sitting thereby decreasing physical inactivity time. The Gentle Jogger does not require multitasking and can be self-administered while watching television, reading, operating a computer and dining, etc. In another study of 26 seated volunteers, mean age 44 years SD 15, BMI 28 SD 5, Gentle Jogger increased METS 15% with no individual exceeding 1.5 METS [26].

Adults spend more than 50% of the waking hours sitting [27]. In diabetic subjects, Fritschi et al. using CGM found a significant correlation between daily time spent in physical inactivity behavior and time spent in hyperglycemia [28]. The effects of breaking up sitting time while continuously monitoring glucose and periods of hyperglycemia were studied by Duvivier et al. in a randomized cross over trial of 19 type 2 diabetics [18]. They studied sitting (14 hr/day) and compared it to a “Sit Less” intervention (replacing sitting with 2.5 hr of standing and 2.2 hr of light walking). After 4 days of Sit Less, they found a 30% reduction of 24 hr AUC and 40% reduction in time in hyperglycemia (time in glucose > 180 mg/dl) compared to baseline. These findings are similar to our study wherein at day 4 of JD we found a 13% decrease in AUC and 46% reduction in time spent in hyperglycemia compared to BL. Other investigators have shown that even a single bout of low-intensity physical activity significantly reduces time in hyperglycemia and improves insulin sensitivity with a 1 day carryover effect [29, 30]. The above findings all support the notion that breaking up sitting time with standing, light-intensity physical activity or walking, and JD benefit glycemic control.

Dempsey et al. studied 24 inactive type 2 diabetic subjects with CGM who were randomized to 7 hr of uninterrupted sitting (SIT), light-intensity walking (LW), or sitting plus 3 min bouts of walking every 30 min, and 3 min of simple resistance activities (calf raises, half squats, gluteal contraction, and knee raises) every 30 min (SRA), each separated by 6-14-day washout period. They found that mGlu and AUC, as well as time in hyperglycemia (glucose > 180 mg/dl), were significantly decreased by both LW and SRA. They found a 23% and 25% decrease from BL for LW and SRA in mGLU, a 24% and 25% from BL for LW and SRA in AUC, and 57% decrease for time in hyperglycemia from BL for both LW and SRA. Additionally, the latter were also reduced during sleeping time [15]. Our findings in diabetic subjects qualitatively agree with theirs. We found that mGlu, AUC, and time in hyperglycemia decreased from baseline by 16%, 16%, and 14%, respectively, after JD. Dempsey et al. used 36 min of cumulative LW or SRA in a 24 hr period in contrast to our study which used 90 min cumulative JD. Despite the latter time differences, the JD intervention was effective in decreasing baseline mGlu, AUC, and 24 hr glucose SUM.

Karstoft et al. studied 14, type 2 diabetic subjects using CGM in a cross over design of 2 interventions short-term interval walking training (IWT, alternating 3 min of fast with 3 min of slow walking for 60 min daily) or continuous walking training (CWT, speed aimed at 73% of peak oxygen consumption) or control (no exercise intervention). Each intervention lasted for 2 weeks with a 4-8-week washout between interventions. These investigators found a significant decrease in mean glucose and % of time in hyperglycemia (glucose > 180 mg/dl) in the IWT group [31]. Unlike our study, this intervention of IWT lasted for 2 weeks. Their mean difference for the decrease in 24 hr mGlu was 0.7 mmol/l (12.6 mg/dl) and a reduction of 9.5% in time in hyperglycemia. Our data for 7 days of JD usage showed a 26 mg/dl difference between BL and JD7 (157 vs. 131 mg/dl). Further, there was a reduction of 55% in time spent in hyperglycemia (BL 591 min vs. JD7 262 min in 24 hr).

Reduction of time spent in hyperglycemia is important, since microvascular and macrovascular complications and proinflammatory phenotype in diabetes have been shown from chronic sustained hyperglycemia and acute glycemic fluctuations [20, 32]. While there are strong grounds to continue to emphasize importance of regular aerobic and muscle-strengthening activities to prevent and manage type 2 diabetes, JD offers a solution to negate the adverse glycemic effects of high amounts (i.e., hours) of sitting in the adult population. Ozawa et al. reported a nonportable (weight 40 kg) passive movement device in a small group of healthy subjects and patients with type 2 diabetes. This device mechanically induced muscular contractions at a rate of 96 per minute around the knee joint. Using a euglycemic clamp, they demonstrated increased glucose uptake but did not report any values of daily glycemic control [33].

As Dempsey et al. have emphasized, there needs to be a whole-day approach that includes more movement and less sitting, as opposed to one or the other [21]. The promotion of more movement while sitting as accomplished with JD is particularly relevant for those individuals who are unable or reluctant to participate in structured exercise. The current study documents the effectiveness of glycemic control using self-administered JD. Long-term trials are needed to ascertain whether JD as a standalone intervention can prevent type 2 diabetes in healthy subjects and reduce insulin and diabetic medications in type 2 diabetic patients.

In the present study, there was a decrease in 24 hr mGlu and %CV in healthy subjects during usage of JD. To our knowledge, there are no reports using CGM in healthy, nondiabetic subjects that document changes in glycemic control due to an intervention. The latter is not surprising since healthy subjects are not expected to have major variability in glycaemia during a 24 hr period. Possibly, our “healthy” subjects had increased physical inactivity time prior to the study and that JD intervention by decreasing such physical inactivity time had its effect on glycemic variability. Further, we cannot exclude that some of the subjects may have been “prediabetic,” based on their medical history but we have no reason to believe such to be the case.

## 5. Study Limitations and Conclusions

There are limitations which must be acknowledged in this pilot study. Our sample size was modest but most studies cited in this paper ranged from 9 to 30 subjects. We did not control for diet, mealtimes, activities of daily living (including exercise time if any), sleep times, or medication dosing, since our intent was to study these subjects under real-world conditions without changing behavior except for daily use of JD. We did not control for BMI; however, post hoc analysis of our data excluding 2 obese subjects in each group was consistent in significant differences with our entire group findings. We also did not control for the number of steps in place used daily on the JD; however, we do know that each subject used the device for at least 10,000 steps in place per day, with a median number of steps of 15,000 and 16,000 steps/day in healthy subjects and diabetics, respectively, based upon our measures of compliance. Our study did not address compliance with long-term usage of the JD and was not powered “a priori,” due to the uncertainty of magnitude of effect. A much larger study with at least 50 type 2 diabetic subjects would be needed based on the current effect size on glucose. Notwithstanding these limitations, in a real-world setting and using CGM, our data are consistent in showing a significant decrease in 24 hr, mGlu, SUM of all glucose measurements, area under the curve, and time spent in hyperglycemia in type 2 diabetics, and a significant decrease %CV, mGlu, and SUM in healthy subjects.

Gentle Jogger is a portable passive movement device which reduces physical inactivity time and positively impacts 24 hr glycemic control. This device can be self-administered in the seated and supine postures. It can be applied to those individuals with physical or cognitive impairments who are nonambulatory. It is applicable as a preventive technology, as a standalone device, or in conjunction with insulin or diabetic therapies.

## Figures and Tables

**Table 1:** Participants characteristics.

No	Sex	Age	Diabetic/healthy	Medications	BMI
1	F	58	Diabetic	Lisinopril, B complex, metformin, insulin	29.3
2	F	51	Diabetic	Insulin, metformin	28.8
3	M	44	Diabetic	Insulin, levothyroxine, vitamin B_12_, Truvada, magnesium	24.8
4	F	73	Diabetic	Atorvastatin, losartan, synthroid, Jentadueto, clonazepam, Brilinta, famotidine, Wellbutrin, pantoprazole	27.8
5	M	62	Diabetic	Metformin	29.7
6	F	76	Diabetic	Synthroid, Victoza	29
7	F	64	Diabetic	Tradjenta, metformin	44.8
8	F	44	Diabetic	Glipizide, gemfibrozil, metformin, aspirin	26.2
9	M	62	Diabetic	Glimepiride, metformin, lisinopril	36.2
10	M	66	Diabetic	Insulin, potassium, atorvastatin, metoprolol, lisinopril, amlodipine, pantoprazole, clopidogrel, bumetanide	24
	6F4M				
*Mean*		*60.0*			*30.1*
*SD*		*10.9*			*6.2*
11	M	63	Healthy	N/A	28.9
12	M	32	Healthy	N/A	27.5
13	F	53	Healthy	N/A	31.8
14	F	32	Healthy	N/A	18.5
15	F	28	Healthy	N/A	22.9
16	M	31	Healthy	N/A	20.3
17	F	28	Healthy	N/A	28.2
18	F	40	Healthy	Melatonin	25.4
19	F	45	Healthy	N/A	26.7
20	M	61	Healthy	N/A	29.6
21	M	73	Healthy	Losartan, levothyroxine, L-carnitine	31.9
	6F5M				
*Mean*		*44.2* ^∗^			*26.5*
*SD*		*16.0*			*4.4*

Study participant characteristics list sex, age, participant category (type 2 diabetic or healthy) current medications, and calculated body mass index (BMI). There were 6 females and 4 males in the type 2 diabetic group with a mean age of 60.0 (10.9) and mean BMI 30.1 (6.2). In healthy subjects, there were 6 females and 5 males, mean age 44.2 [16]^∗^ and BMI 26.5 (4.4). ^∗^*p* < 0.02 healthy vs. diabetics. Data are expressed as the mean (SD, standard deviation).

**Table 2:** Twenty-four-hour average glycemia and indices in healthy subjects at baseline, during, and after Gentle Jogger jogging device (JD).

	BL	JD2	JD3	JD4	JD5	JD6	JD7	Post JD1	Post JD2
*mGlu (mg/dl)*	92.7	82.4^∗^	84.4^∗^	83.6^∗^	83.3^∗^	82.8^∗^	81.0^∗^	85.9	85.24
SD	6.6	6.7	7.5	7.5	7.9	8.4	10.2	11.3	9.213
*SUM (mg/dl)*	8929	7932^∗^	8126^∗^	8046^∗^	8017^∗^	7968^∗^	7801^∗^	8262	8205
SD	632.7	642.1	705.5	708.3	748.1	789.4	979.6	1073	873.9
*%CV*	21.1	11.9	14.3	15.4	12.7^∗^	13.2^∗^	12.4^∗^	17.2	18.4
SD	7.7	3.4	5.3	6.7	3.3	5.8	4.3	5.6	5.2
*AUC*	1802	1630^∗^	1653^∗^	1733	1626^∗^	1666	1629	1723	1687
SD	236.2	164.3	193.7	185.3	156.5	180.9	228	234.1	254.2
*% time in range (TIR*, *glucose* 70-180 *mg/dl)*									
Mean	93.8	93.6	94.2	95.9	96.5	93.0	87.9	93.6	91.1
SD	9.4	12.4	11.5	8.4	8.4	14.9	26	13.2	16.6
*% time below range (TBR*, *glucose* < 70 *mg*/*dl*)									
Mean	5.2	5.4	6.9	6.5	5.9	5.7	11.1	7.7	9.2
SD	9.9	12.2	11.0	11.0	11.6	17.0	25.0	13.3	15.9

Values for twenty-four-hour mean glucose (mGlu, mg/dl), sum of 24 hr glucose (SUM mg/dl), coefficient of variation (%CV), and 24 hr area under the glucose curve (AUC), in healthy subjects. % time in range (TIR, glucose between 70 and 180 mg/dl, % time below range (TBR, glucose < 70 mg/dl). Data are expressed as the mean (SD, standard deviation). The time points are 24 hr prior to operation of JD (BL), after 2, 3, 4, 5, 6, and 7 days of JD (JD2, JD3, JD4, JD5, JD6, JD7), and 24 and 48 hr after discontinuation of JD (Post JD1, Post JD2). Statistical significance ^∗^ < *p* < 0.01 vs. BL.

**Table 3:** Twenty-four-hour average glycemia and indices in diabetic subjects at baseline, during, and after Gentle Jogger jogging device (JD).

	BL	JD2	JD3	JD4	JD5	JD6	JD7	Post JD1	Post JD2
*mGlu (mg/dl)*	157	130.4^∗^	130.6^∗^	131.7^∗^	127.1^∗^	130.8^∗^	130.7^∗^	140.1^∗^	144.2
SD	45.9	35.0	41.1	41.6	33.6	31.3	31.5	42.1	38.6
*SUM (mg/dl)*	15071	12517^∗^	12542^∗^	12645^∗^	12201^∗^	12555^∗^	12547^∗^	13451^∗^	13839
SD	4416	3363	3947	3997	3224	3009	3029	4044	3702
*%CV*	25.56	18.5	22.45	21.2	20.95	18.03	18.79	22.48	25.78
SD	9.2	5.9	7.9	6.6	9.0	7.0	6.7	7.9	8.7
*AUC*	3129	2665^∗^	2659^∗^	2689^∗^	2589^∗^	2671^∗^	2566^∗^	2758^∗^	2829
SD	946.2	734.4	831.8	850.5	661.1	673	602.9	861.9	840.9
*% time above range (TAR*, *glucose* 181-250 *mg/dl)*									
Mean	28.0	7.9^∗^	9.7^∗^	8.3^∗^	5.8^∗^	3.6^∗^	5.9^∗^	11.2	19.1
SD	17.4	9.8	13.4	13.7	8.3	5.9	6.5	13.9	16.9
*% time in range (TIR*, *glucose* 70-180 *mg/dl)*									
Mean	59.0	77.6^∗^	71.0	74.1^∗^	77.39^∗^	81.5^∗^	81.3^∗^	70.1	66.7
SD	32.9	30.0	33	32.9	29.7	30.5	30.1	34.4	29.8
*% time below range (TBR*, *glucose* < 70 *mg*/*dl*)									
Mean	3.8	1.8	5.3	3.7	3.9	2.2	0.85	0.8	3.7
SD	5.5	3.3	7.8	9.5	8.6	6.6	1.3	1.1	9.2

Values for twenty-four-hour mean glucose (mGlu, mg/dl), sum of 24 hr glucose (SUM mg/dl), coefficient of variation (%CV) and 24 hr area under the glucose curve (AUC), in type 2 diabetics. % time above range (TAR, glucose 181-250 mg/dl), % time in range (TIR, glucose between 70 and180 mg/dl, and % time below range (TBR, glucose < 70 mg/dl). Data are expressed as mean (SD, standard deviation). The time points are 24 hr prior to operation of JD (BL), after 2, 3, 4, 5, 6, and 7 days of JD (JD2, JD3, JD4, JD5, JD6, JD7), and 24 and 48 hr after discontinuation of JD (Post JD1, Post JD2). Statistical significance ^∗^ < *p* < 0.01 vs. BL, ^†^*p* < 0.05 vs. BL.

## Data Availability

The data used to support the findings of this study are available from the corresponding author upon request.

## Supplementary Materials

Supplementary Materials This file contains Figure S1, which is the study flow diagram based on CONSORT Criteria and Supplemental Table 1, which contains twenty-four-hour average glycaemia measurements and indices, in both healthy and diabetic subjects excluding obese subjects, at baseline, during and after use of the jogging device.
