# Supplementary material for: Portable Gentle Jogger Improves Glycemic Indices in Type 2 Diabetic and Healthy Subjects Living at Home: A Pilot Study
Source: J Diabetes Res. 2020 Jan 21;2020:8317973. doi: 10.1155/2020/8317973 (PMC7081036; doi:10.1155/2020/8317973)
Supplement: Supplementary Materials — This file contains Figure S1, which is the study flow diagram based on CONSORT Criteria and Supplemental Table 1, which contains twenty-four-hour average glycaemia measurements and indices, in both healthy and diabetic subjects excluding obese subjects, at baseline, during and after use of the jogging device. [file 8317973.f1.pdf]

**Supplemental Material File Figure 1S**

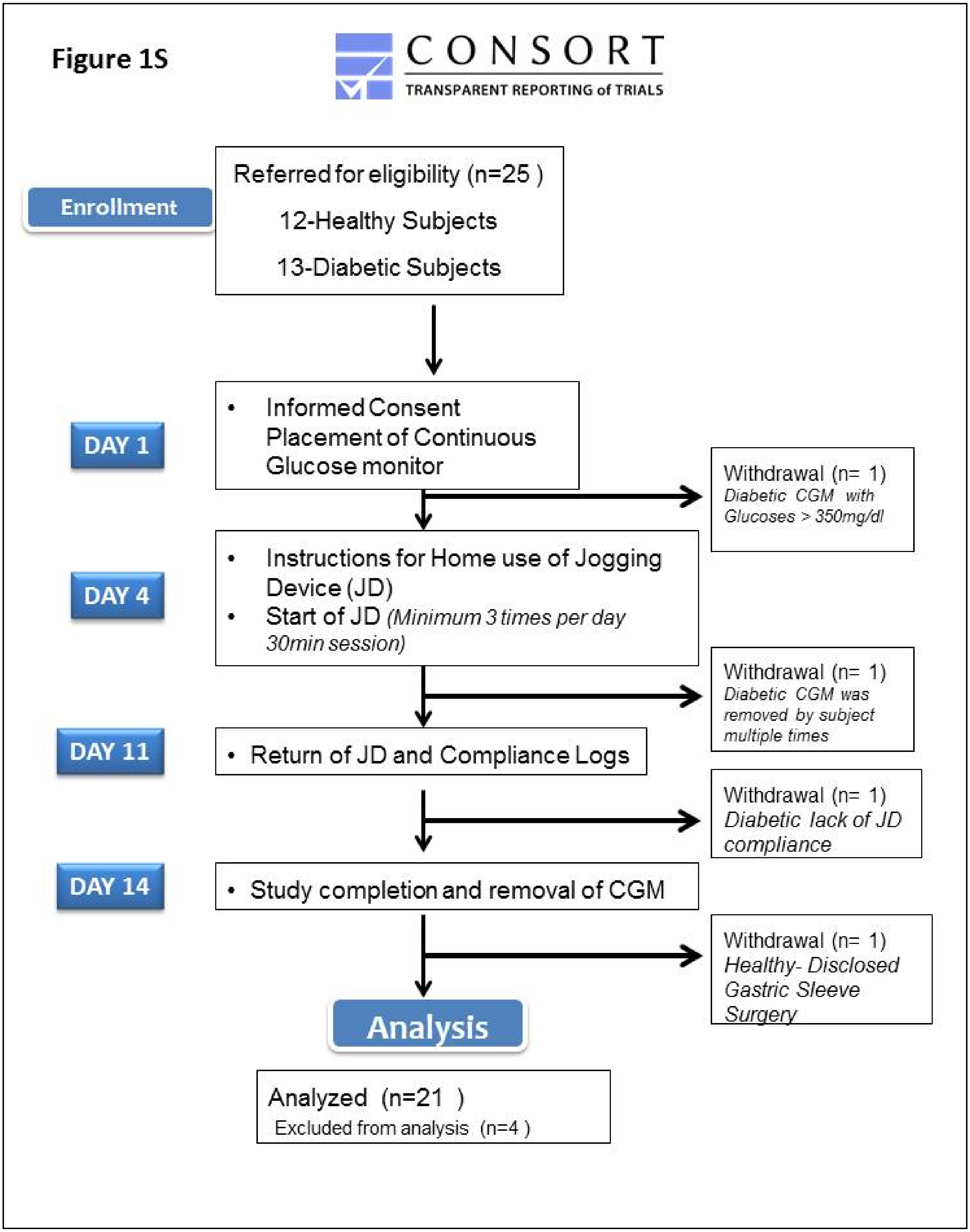

*Legend: Study flow diagram based on CONSORT Criteria. There were 25 subjects recruited and 21 subjects analyzed. The four  subjects not analyzed were due to; one subjects whose baseline glucose exceeded  350mg/dl, two  noncompliance with the use of JD,  or continuous glucose monitoring device (CGM), and a “healthy subject” who later disclosed she had a gastric sleeve placed.*

**Supplemental Table 1**

***Twenty-Four Hour Average Glycaemia and Indices in Healthy and Diabetic Subjects at Baseline, During and After Jogging Device(JD) Excluding Obese Subjects***

| | BL | JD2 | JD3 | JD4 | JD5 | JD6 | JD7 | Post JD1 | Post JD2 |
|---|---|---|---|---|---|---|---|---|---|
| **HEALTHY** | | | | | | | | | |
| mGlu(mg/dl) | 91.4 | 80.8* | 82.8* | 81.9* | 81.8* | 81.5* | 79.1* | 83.2 | 83.6 |
| SD | 6.6 | 6.0 | 7.3 | 7.2 | 8.1 | 8.6 | 10.3 | 10.0 | 9.3 |
| | | | | | | | | | |
| SUM(mg/dl) | 8807 | 7786* | 7974* | 7885* | 7877* | 7849* | 7624* | 8006 | 8050 |
| SD | 631.5 | 583.6 | 693.2 | 676.1 | 758 | 818.2 | 985.7 | 953.7 | 883.8 |
| | | | | | | | | | |
| %CV | 20.3 | 11.5* | 14.9 | 14.2 | 12.1* | 12.8* | 11.9* | 16.5 | 18.3 |
| SD | 6.2 | 3.4 | 4.6 | 4.9 | 2.8 | 5.1 | 2.7 | 5.7 | 5.1 |
| | | | | | | | | | |
| AUC | 1832 | 1605* | 1618* | 1728 | 1620* | 1639* | 1599* | 1659 | 1644 |
| SD | 232.7 | 170.8 | 198.2 | 206.7 | 164.1 | 180.1 | 233.8 | 206.5 | 257.7 |
| | | | | | | | | | |
| **DIABETICS** | | | | | | | | | |
| mGlu(mg/dl) | 157.5 | 130.7* | 131.8* | 133.6* | 129.4* | 132.6* | 130.9* | 138.7* | 142.9 |
| SD | 52.1 | 38.6 | 45.6 | 46.4 | 37.0 | 35.0 | 35.5 | 47.6 | 43.4 |
| | | | | | | | | | |
| SUM(mg/dl) | 15116 | 12552* | 12655* | 12826* | 12424* | 12734* | 12571* | 13313* | 13720 |
| SD | 5006 | 3703 | 4378 | 4455 | 3553 | 3362 | 3409 | 4573 | 4164 |
| | | | | | | | | | |
| %CV | 25.4 | 19.9 | 20.1 | 21.6 | 21.0 | 15.5 | 18.3 | 22.8 | 27.1 |
| SD | 10.2 | 5.5 | 6.7 | 6.8 | 10.2 | 4.4 | 7.5 | 8.9 | 9.2 |
| | | | | | | | | | |
| AUC | 3153 | 2692 | 2677* | 2770 | 2634* | 2717 | 2593* | 2737* | 2846 |
| SD | 1071 | 810.4 | 941.3 | 921.9 | 741.8 | 754.2 | 674.7 | 975.9 | 950 |
| | | | | | | | | | |

Legend: Values for twenty-four hour mean Glucose (mGlu, mg/dl), sum of 24 hr glucose (SUM mg/dl), Coefficient of Variation (%CV) and 24 hr area under the glucose curve (AUC), in Healthy and Type 2 Diabetics. Data are expressed as mean (SD, standard deviation). Excludes obese subjects (BMI>=30) for both Healthy (n=9) and Diabetics (n=8). Statistical significance *< p< 0.01 vs BL.
